# Identifying self-assessed competencies and areas for improvement within community pharmacist-preceptors support during pre-registration training

**DOI:** 10.1186/s12909-018-1413-x

**Published:** 2018-12-11

**Authors:** Margita Držaić, Ingrid Kummer, Iva Mucalo, Andreia Bruno, Maja Ortner Hadžiabdić

**Affiliations:** 1 City Pharmacies Zagreb, Zagreb, Croatia; 2 Faculty of Pharmacy and Biochemistry, University of Zagreb, Zagreb, Croatia; 3 Faculty of Pharmacy and Pharmaceutical Sciences, University of Monash, Melbourne, Australia; 4 Faculty of Pharmacy and Biochemistry, University of Zagreb, Ante Kovačića 1, 10 000 Zagreb, Croatia

**Keywords:** Competency, Pharmacist-preceptor, Community pharmacy, Croatian competency framework, Continuing professional development

## Abstract

**Background:**

Competency frameworks that prompt personal and professional development have become an important component of lifelong learning; they are driven by healthcare professionals’ need for development and professional recognition. This study aimed to evaluate the self-assessed competencies of community pharmacist-preceptors by using Croatian Competency Framework (CCF) and to identify competencies to be improved. The secondary aim was to explore the association between community pharmacists’ characteristics (i.e. age, education etc.) and self-assessed competency performance.

**Methods:**

The study subjects were community pharmacist-preceptors who provide support to and mentor student trainees enrolled in pre-registration training for pharmacy students. At the beginning of their mentorship, the pharmacist-preceptors assessed their competencies on a four-point Likert scale by using the Croatian Competency Framework (CCF), a validated tool for assessment and self-assessment of community and hospital pharmacists. Data were collected via e-mail in the period from October 2015 to April 2016.

**Results:**

Of the 260 community pharmacists approached, final analysis included 223 respondents. The response rate was 85.8%. Community pharmacist-preceptors assessed themselves as the most competent in competencies pertaining to the cluster “Organization and management competencies” (M = 3.64, SD = 0.34), while they considered themselves as the least competent in the competencies pertaining to the cluster “Pharmaceutical public health competencies” (M = 2.75, SD = 0.77). Younger pharmacists with a postgraduate qualification who worked for large pharmacy chains in the capital city area and who had been in their current posts for a shorter period perceived themselves to be more competent.

**Conclusion:**

This research represents the first analysis of the CCF in practice and identifies community pharmacist-preceptor competencies that require improvement. Consequently, areas for additional professional education were defined. Implementing modalities to measure and support development of preceptors’ competences is essential for improvement of student training programmes.

## Background

In the last few decades, intense changes in the pharmacy profession have been witnessed, which have led to the development of competencies in pharmacy. These have been imposed as basic prerequisites for the provision of pharmaceutical care, and for improving therapeutic outcomes and patient’s quality of life. Therefore, competency frameworks that prompt personal and professional development have become an important component of lifelong learning; they are driven by healthcare professionals’ need for development and professional recognition.

Up till now, the applicability of competency frameworks for the pharmacy profession has been evidenced by its many uses, such as supporting pharmacists in their continuing professional development (CPD) [1–3], assisting CPD providers in developing educational materials or courses for pharmacists [4], and assisting educators at Universities in evaluating and developing curricula and training programmes for pharmacy students [5, 6] — this list not being exhaustive.

Maintaining competence throughout a career is a lifelong challenge. As healthcare professionals have not always been sufficiently motivated to pursue new knowledge and skills, professional associations and authorities started developing formal lifelong learning systems [7]. This article addresses pharmacy practice and education in Croatia, where the traditional system of lifelong learning is based on continuing education (CE) and linked with mandatory registration and re-registration. This system was implemented by the Croatian Chamber of Pharmacists in 1995. Like many pharmacists globally, Croatian pharmacists are required to collect a minimum number of points within a defined period of time (30 points within 6 years) through participation in various development activities (lectures/presentations, workshops, conferences, among others). Recently, there has been substantial debate about the limited effects of formal CE activities on pharmacists’ performance and the need for a shift towards competency-based CPD. Therefore, the development of a national competency framework for pharmacists was recognised as highly important. It could help to bridge the gap between traditional pharmacy education and the ever-changing demands of modern healthcare systems [8]. The first national competency framework for pharmacists in Croatia (CCF) was introduced in 2015, and, eventually, the University of Zagreb’s (UoZ) Faculty of Pharmacy and Biochemistry implemented it in the pre-registration training of pharmacy students. The new pre-registration training programme, based on CCF, was developed and introduced in 2016. Additionally, CCF is now used at the UoZ Faculty of Pharmacy and Biochemistry for evaluation and self-evaluation of both students and mentors during the pre-registration training. Therefore, for the first time, CCF was tested in practice, and substantial data has been generated on the use of CCF among Croatian pharmacists.

The aim of this study was to evaluate the self-assessed competencies of community pharmacist-preceptors by using the CCF and to identify competencies that need improving in order to enhance the quality of pharmacists’ interventions in community pharmacies. Additionally, this study explores the association between community pharmacists’ characteristics and their self-assessed competency performance. Our hypothesis was that differing levels of education related to age and experience could cause substantial differences in the competencies of the different groups studied.

## Methods

### Sample, setting and data analysis

Of the 260 Croatian community pharmacists approached, 223 respondents participated in the observational study conducted by the UoZ Faculty of Pharmacy and Biochemistry. They were provided with the education needed for supporting and mentoring student trainees at the university department. The invitation was announced through the Croatian Chamber of Pharmacists web site 2 months prior to the commencement of the training programme. The pre-requirements for participation were: (1) at least 2 years of experience as a registered community pharmacist; and (2) completion of the training programme for preceptors provided by the UoZ Faculty of Pharmacy and Biochemistry. All volunteering pharmacists who were interested in becoming mentors attended a one-day training programme held by the UoZ Faculty of Pharmacy and Biochemistry’s Centre for Applied Pharmacy, where they were introduced to the competency-based pre-registration programme and mentors’ duties and responsibilities. The training programme consisted of lectures and workshops on the principles of adult learning, workplace education and work-based learning, and the practical uses of CCF. The content covered, among other things, the principles of the needs-based education model, the concept of competency frameworks, and an introduction to CCF along with guidance on use. This ensured that all the participating pharmacists gained a basic understanding of the frameworks and the self-assessment approach. This setting allowed for the creation of a sample that was representative of Croatian pharmacists; participants came from different regions of the country, and they also worked in different types of pharmacies (private or state owned; chains or individual pharmacies, etc.).

Following the mentors’ training, each mentor was invited via e-mail to conduct a self-evaluation by using the CCF. To ensure a high response rate, a mixed-mode approach of data collection that combined two rounds of e-mailed survey instrument with a telephone reminder was employed. Respondents were asked to self-evaluate their competencies on a four-point Likert scale, based on how frequently a certain behaviour was demonstrated in their everyday practice: rarely (0 to 24% of the time); sometimes (25 to 50% of the time); usually (51 to 84% of the time); or consistently (85 to 100% of the time). If behaviour was not relevant to a respondent’s practice, it was categorised as “not-applicable.” Each self-evaluation, comprising of 4 competency clusters, 17 competencies, and 96 behavioural statements, was recorded into an Excel sheet, which had been previously prepared for the CCF evaluation. The development of the CCF tested in this study has been already described in detail in the literature [8]. It originates from the Global Competency Framework (GbCF) developed by International Pharmaceutical Federation Education (FIP*Ed*) [9] and was adapted through the rigid process of translation, consensus development, and validation [8]. The adapted and validated CCF yielded the following four clusters: “Pharmaceutical Public Health Competencies,” “Pharmaceutical Care Competencies,” “Organization and Management Competencies,” and “Personal and Professional Competencies.” Data collection took place between October 2015 and April 2016.

Sociodemographic characteristics were obtained from a mentors’ database, which included participants’ information such as gender, educational level, years of practice in a community pharmacy, and type of community pharmacy (individual pharmacy or chain). Ethical approval was waived by the Ethical Committee of the Faculty of Pharmacy and Biochemistry, University of Zagreb.

Data were anonymised and transferred from the Excel database on to a Statistical Package for Social Sciences database (SPSS, IBM Corp., Armonk, NY, USA) for analysis.

The descriptive analysis of the study population’s characteristics included frequency distribution for the qualitative variables and measures of central tendency and dispersion for the quantitative variables. Normality of variable distribution was assessed through the Kolmogorov-Smirnov test. Independent t-test and One-Way ANOVA with post hoc Tuckey HD test were used to analyse the differences in performance levels between two or more groups of community pharmacists with different sociodemographic characteristics. Welch test was used instead of ANOVA when the assumption of homogeneity of variances was violated (*p* < 0.05). Statistical significance was placed on any *p* value that was less than 0.05 for a two-sided test.

## Results

Overall, 223 pharmacists from 168 community pharmacies responded to the questionnaire (response rate: 85.8%) and were subsequently included in the final analysis.

Out of 223 participants of the study, 48.0% responded following the first e-mail invitation, 20.6% following the second e-mail invitation and 31.4% following the telephone follow-up.

Participants corresponded to approximately 10% of the pharmacists in the country. Pharmacists enrolled in the study were predominantly female 91.5% (*n* = 204), with the majority (43%) belonging to the 36–45 age group. The respondents’ median age was 41 years, with ages ranging from 27 to 65 years. A total of 31.8% of respondents had been in practice for 10 years or less, 42.6% had been in practice for 11–20 years, and the remainder (25.6%) had been practicing for more than 21 years. Around 7% of pharmacists (*n* = 16) worked in independent pharmacies, and the remainder (93.3%) were employed in large private or state-owned pharmacy institutions. Out of all the respondents, 10.8% (*n* = 24) held a postgraduate qualification, out of which 50% (*n* = 12) were in the clinical pharmacy field. The main characteristics of the study participants are shown in Table 1.

**Table 1:** Demographic Characteristics of Community Pharmacists

Variable		n	%
Gender	Male	19	8.5
	Female	204	91.5
Age (years)	median (range)	41 (27–65)	
	<35	59	26.5
	36–45	96	43.0
	46–55	45	20.2
	>56	23	10.3
Work experience in community pharmacy (years)	median (range)	14 (2–40)	
	< 10	71	31.8
	11–20	95	42.6
	21–30	43	19.3
	31–40	14	6.3
Location of pharmacy	City of Zagreb	103	46.2
	rest of the Croatia	120	53.8
Education	Master degree-level pharmacists	199	89.2
	Master degree-level pharmacists with additional postgraduate education	24	10.8
Type of community pharmacy	single pharmacy	16	7.2
	pharmacy institution	207	92.8
Pharmacy ownership	single private pharmacy	8	3.6
	private pharmacy institution	123	55.2
	state-owned pharmacy institution	84	37.7
	pharmacy under private lease	8	3.6
Size of pharmacy chain	single pharmacy	16	7.2
	medium (2–10 pharmacies)	71	31.8
	large (> 10 pharmacies)	136	61.0

The most prevalent areas for improvement centred around two competency clusters: “Pharmaceutical Public Health Competencies” and “Pharmaceutical Care Competencies.” Overall, respondents in this study perceived themselves as the most competent in the “Organization and Management Competencies” cluster (3.64 ± 0.336), whereas the lowest performance was noted in the “Pharmaceutical Public Health Competencies” cluster (2.75 ± 0.769) (Table 2). From the 96 behaviours analysed, community pharmacists demonstrated the best performance in the behavioural statements pertaining to the “Dispensing medicines and medical devices” competency within the “Pharmaceutical Care Competencies” cluster: “Accurately dispense prescribed medicines” (3.97 ± 0.162), “Accurately dispense medical devices based on certification of medical device” (3.97 ± 0.175), and “Establish accuracy and legality of prescriptions and certifications of medical devices and validate them appropriately” (3.96 ± 0.219) (Table 3). The five behaviours with the lowest performance were in the “Assurance of safe use of medicines” competencies within the “Pharmaceutical Care Competencies” cluster: “Regularly report medication errors to internal Committees/appropriate authorities, if possible” (1.66 ± 0.192), “Regularly monitor and be up to date with medication error reports” (1.83 ± 0.945), and “Document medication errors in internal medication error reporting systems” (2.01 ± 1.070) (Table 3).

**Table 2:** Self-Assessed Competencies’ Mean Scores

Cluster and Competency Code	Cluster	Competency	Mean Score	SD^a^
KK1	Pharmaceutical Public Health Competencies		2.75	0.769
K1.1.		Health promotion	2.75	0.769
KK2	Pharmaceutical Care Competencies		3.29	0.375
K2.1.		Assessment of medicines and medical devices	3.41	0.513
K2.2.		Compounding of magistral and galenic preparations	3.63	0.436
K2.3.		Dispensing medicines and medical devices	3.68	0.274
K2.4.		Patient consultation	3.13	0.529
K2.5.		Assurance of safe medicines use	2.62	0.685
KK3	Organization and Management Competencies		3.64	0.336
K3.1.		Organization	3.71	0.470
K3.2.		Finance management	3.69	0.483
K3.3.		Human resources management	3.36	0.692
K3.4.		Workplace management	3.70	0.403
K3.5.		Procurement and supply chain management	3.74	0.272
KK4	Personal and Professional Competencies		3.41	0.405
K4.1.		Communication skills	3.70	0.397
K4.2.		Continuing Professional Development (CPD)	3.34	0.554
K4.3.		Legal and regulatory practice	3.08	0.682
K4.4.		Professional and ethical practice	3.77	0.393
K4.5.		Improvement of service and quality assurance	3.03	0.751
K4.6.		Personal development	3.54	0.537

^a^*SD* standard deviation

**Table 3:** Group Differences in Cluster/Competency Self-Assessed Mean Scores

Cluster/Competency	Age Group			Education		Location of Pharmacy		Size of Pharmacy Chain	
	≤35 years	36–50 years	> 50 years	MPharm	MPharm with additional postgraduate education	Zagreb	rest of Croatia	≤10 pharmacies	> 10 pharmacies
Pharmaceutical Public Health Competencies	2.78	2.73	2.78	2.71*	3.12	2.77	2.74	2.70	2.79
Health promotion	2.78	2.73	2.78	2.71*	3.12	2.77	2.74	2.70	2.79
Pharmaceutical Care Competencies	3.38	3.25	3.29	3.29	3.37	3.33	3.27	3.24	3.33
Assessment of medicines and medical devices	3.48	3.39	3.40	3.40	3.56	3.44	3.39	3.38	3.44
Compounding of magistral and galenic preparations	3.66	3.62	3.64	3.62	3.71	3.70*	3.57	3.521	3.70***
Dispensing medicines and medical devices	3.72	3.66	3.68	3.68	3.73	3.68	3.68	3.64	3.71
Patient consultation	3.20	3.09	3.13	3.14	3.09	3.13	3.13	3.12	3.14
Assurance of safe medicines use	2.85##	2.51##	2.61	2.60	2.77	2.68	2.58	2.54	2.67
Organization and Management Competencies	3.62	3.67	3.61	3.64	3.68	3.64	3.65	3.62	3.66
Organization	3.69	3.72	3.70	3.66	3.83	3.67	3.74	3.77	3.67
Finance management	3.61	3.75	3.62	3.67	3.80	3.67	3.67	3.65	3.71
Human resources management	3.31	3.39	3.35	3.36	3.33	3.35	3.37	3.30	3.40
Workplace management	3.69	3.71	3.67	3.70	3.70	3.72	3.68	3.65	3.73
Procurement and supply chain management	3.75	3.76	3.69	3.74	3.75	3.75	3.74	3.72	3.76
Personal and Professional Competencies	3.47	3.38	3.41	3.64	3.68	3.44	3.38	3.35	3.45
Communication skills	3.71	3.71	3.69	3.70	3.69	3.70	3.70	3.65	3.74
Continuing Professional Development (CPD)	3.40	3.30	3.37	3.32	3.44	3.44**	3.25	3.23	3.40*
Legal and regulatory practice	3.06	3.06	3.17	3.09	3.04	3.09	3.08	3.01	3.13
Professional and ethical practice	3.82	3.74	3.75	3.76	3.84	3.79	3.74	3.77	3.76
Improvement of service and quality assurance	3.12	2.99	3.00	3.03	2.98	3.01	3.04	2.97	3.06
Personal development	3.71#	3.48#	3.47	3.53	3.58	3.61	3.47	3.47	3.58

- difference is statistically significant at the *p* ≤ 0.05 (*), *p* ≤ 0.01 (**) and *p* ≤ 0.005 (***) level (exact, 2-tailed)

- difference is statistically significant at the *p* ≤ 0,05 (#) and *p* ≤ 0,01 (##) (One-way ANOVA)

In general, there was more variation in the means for the competencies in the “Pharmaceutical Care Competencies cluster” than in any other competency clusters; the mean responses ranged from “rarely” and “sometimes” for the “Assurance of safe use of medicines” and “Patient consultation” competencies to “usually” and “consistently” for the “Dispensing medicines and medical devices” competency. The highest variance in pharmacists’ behaviour was observed in the following areas: “Ascertain the quality of galenic preparation” (1.386), “Confirm the identity and quality of entry/raw materials” (1.353), and “Obtain patient consent when necessary” (1.210). The least variation was found within the behavioural statements pertaining to the “Organization and Management Competencies cluster.” Nevertheless, the respondents perceived themselves to be less competent at managing human resources, as opposed to managing procurement and supply chains, performing organization tasks, and managing finances. There was slightly more variation in the means of competencies pertaining to the “Personal and Professional Competencies cluster,” with the mean response for the “Improvement of service and quality assurance” competency being the lowest.

In general, younger pharmacists with a postgraduate qualification who worked for large pharmacy chains (consisting of more than 10 pharmacies) in the Zagreb area and who had been in their current posts for a shorter period of time perceived themselves to be more competent in seven behaviours within the four competency clusters (Table 3). Pharmacists who had been in their current posts for a shorter period of time (less than 15 years) perceived themselves to be more competent in behaviours pertaining to the “Assurance of safe medicines use,” “Procurement and supply chain management,” and “Continuing Professional Development” competencies (*p* < 0.05). Statistically significant differences were observed in the “Assurance of safe medicines use” (F = 4.735, *p* < 0.01) and “Personal development” competencies (F = 5.939, *p* < 0.05) between the different age groups of pharmacists (group 1 - below the age of 35; group 2–36-50 years of age; group 3 - above the age of 50). The highest level of self-assessed competence was observed in the youngest age group. Additionally, pharmacists below the age of 35 assessed their level of competence as higher in behaviours pertaining to the “Patient consultation,” “Assurance of safe medicines use,” “Procurement and supply chain management,” and “Personal development” competencies. Pharmacists who held a postgraduate qualification perceived themselves to be more competent in the “Health promotion” competency in the “Pharmaceutical Public Health Competencies cluster” (*t* = − 2.414, *p* < 0.05). Respondents who worked in larger chain pharmacies in the Zagreb area assessed themselves as better in the “Compounding of magistral and galenic preparations” (*p* < 0.001) and “Continuing Professional Development” competencies (*p* < 0.05).

## Discussion

This study explored the self-assessment of community pharmacist-preceptors and found that pharmacists perceived themselves as the most competent in “Organization and Management” and the least competent in patient oriented behaviours contained in the “Public health” competency cluster and the “Pharmaceutical care” cluster. The deficiency in patient oriented competencies among community pharmacists comes as no surprise, as community pharmacy has traditionally been a profitable business and similar trends have been observed in previous studies. In congruence with previous research, including Stojkov’s 2016 study [10], our results revealed the highest scores and the lowest variation of the behavioural statements in the “Organization and Management” cluster competencies, as expected. Wright and Morgan [11] reported that community pharmacists tended to identify difficulties in demonstrating some of the clinical competencies, while hospital pharmacists reported difficulties in demonstrating management competencies. Indeed, the “Organization and Management” cluster was added into the General Level Framework (GLF) — the first competency framework for early career pharmacists in the UK — only after the original framework was adapted for community pharmacists because it was considered important for community pharmacists’ roles [12]. Furthermore, the high scores identified in this cluster are in accordance with the pharmacists’ role as summarised in the “Seven star pharmacist,” which states that pharmacists must possess management and leadership skills [13]. The participants were aware of the relevance of these skills in their everyday practice.

Additionally, the high scores obtained by pharmacist-preceptors in the “Organization and Management” cluster competencies are of great importance for students involved in the pre-registration training programme. Indeed, education in management, leadership, and organizational skills tends to be deficient during the graduate programme, and the fact that we have competent mentors in this area could compensate for the abovementioned limitation.

On the other hand, pharmacists assessed themselves as less competent in the “Pharmaceutical care competency” cluster, with the lowest scores in the “Assurance of safe medicines use” competency and the highest in the “Dispensing medicines and medical devices” competency. These results coincide with the traditional role of a pharmacist and reflect the slow rate of change in the pharmacy profession. The role of a pharmacist in Croatia is still very much drug-oriented, and dispensing remains the pharmacist’s chief activity; thus, it is no wonder that the participant pharmacists perceived themselves to be most competent in the activities they practiced the most. Moreover, our results also parallel those obtained from the first study on competency assessment conducted in Croatia 7 years ago. In Meštrović et al’s study, community pharmacists demonstrated the best performance in the “Dispensing medicines” competency [14]. It demonstrates that, unfortunately, no significant change has occurred in the last decade.

The lowest self-assessed competency “Assurance of safe medicines use” is a great concern for patient safety and implies the urgent need for substantial changes in health policy as well as pharmacy training and education. This competency consists of six behavioural statements among which “medication error reporting and documentation” attained the lowest assessments due to the lack of a national system or predefined community pharmacy standard operating procedures for medication error reporting. All healthcare professionals in Croatia are obliged to report adverse drug reactions (ADRs) or suspected ADRs to the Agency for Medicinal Products and Medical Devices of Croatia. However, they have no obligation to report medication errors. In recent times, the application form for ADR reporting has been extended, to include a check box to indicate ADRs resulting from medication errors. However, based on self-assessed competencies, it is possible that community pharmacists are not aware of this novelty and still do not report medication errors. Therefore, the appropriate authorities should decide if ticking a check box to report ADRs is a sufficient method for reporting medication errors or if a new separate system should be developed for medication error reporting. Wright and Morgan appropriately commented that, with community pharmacy provided services becoming more complex and the greater chances of causing harm to patients, it should be rethought whether more formal qualifications or regulations may be required for community pharmacists [11].

Studies exploring the association between pharmacists’ characteristics and self-assessed competencies are limited [15]. Therefore, the secondary aim was to identify the differences in self-assessed competencies with regard to pharmacists’ characteristics (age, years of practice, level of education, type of pharmacy, etc.). This was of particular interest because pharmacists practicing in the Croatian community pharmacies tend to possess different educational backgrounds due to the substantial development in pharmacy education during the last 10 years. Therefore, the assumption was that it could result in obtaining different levels of competencies for practicing community pharmacists.

In accordance with previously conducted research [12, 15, 16], younger pharmacists who had been in their current posts for a shorter period of time (less than 15 years) perceived themselves as more competent in behaviours pertaining to the “Patient consultation,” “Assurance of safe medicines use,” and “Continuing Professional Development” competencies, some of which are important for pharmaceutical care provision. This implies that, besides working experience, the education levels or qualifications and CPD are also crucial for competence development. Pharmacy education in Croatia has gone through substantial changes in the last decade, and, in 2005, the four-year graduate programme was extended to a five-year programme in accordance with the Bologna declaration recommendations. This change was supported by the TEMPUS JOINT project “New Pharmacy Curricula-Development and Implementation” (TEMPUS JEP-18028-2003). Prior to curriculum reform, the pharmacy graduate programme was heavily weighted towards science (chemistry in particular), and students remained mainly within the academic setting. Nowadays, an additional practice/clinical focused component has been added in the final year. Pharmacists who were younger than 35 and perceived themselves as more competent in some of the pharmaceutical care competencies graduated from the new pharmacy programme, indicating that the introduction of the new curricula might have had an impact on practicing pharmacists’ competencies. The fact that younger pharmacists assessed their competencies in the “Pharmaceutical Care” cluster significantly higher than their older colleagues could imply that the new generation may have been better prepared for the evolving pharmacist role. This fact also highlights the significance of the new reformed graduate curriculum. This is an important finding and the first evidence of the impact of the new curriculum on pharmacists’ practice, and it implies the need for further exploration of this implication.

Other authors provide alternative explanations for this tendency; Mills et al. suggest that this may be a reflection of younger practitioners’ confidence in their skills, knowledge, and abilities, which may result from their participation in CPD. They also suggest that older pharmacists may be more cautious in their self-assessments [12].

Surprisingly, pharmacists perceived themselves as the least competent in “Public health” competencies. The authors believe that pharmacists have the potential to make greater contributions to public health. The convenient accessibility of community pharmacies enables pharmacists to support public health by promoting healthy lifestyles, teaching patients to prevent or manage chronic diseases better, and improving patients’ adherence to the medicines. Indeed, World Health Organization points out that, globally, community pharmacies have been identified as an easily accessible and cost-effective platform for delivering healthcare services [17]. Eades et al’s systematic review on public health services in community pharmacies found that most pharmacists viewed public health services as an important part of their role but secondary to medicine-related services and that pharmacists’ confidence in public health services provision was, on the whole, average to low [18]. They also suggest that training must aim to increase pharmacists’ confidence in providing these services in order to improve public healthcare services. Confident, well-trained pharmacists will be able to offer public health services more proactively, which is likely to have a positive impact on patient attitudes and health [18]. Interestingly, in this study, only the pharmacists holding postgraduate educational qualifications perceived themselves to be more competent in the “Public health” competency — a tendency that emphasises the impact of clinical pharmacy postgraduate studies on this very important aspect of patient care.

Another factor that emerged in the exploration of the influences on pharmacists’ competence was the type of pharmacy. It was observed that pharmacists employed in pharmacy chains perceived themselves as more competent than those working in individual pharmacies. Pharmacists’ development in the workplace, especially in small pharmacies, was affected by a predominant culture of working in isolation, such work conditions may have had an impact on their competencies. Large pharmacy chains in Croatia usually organize internal training for their employees and assess their pharmacists’ competency and work productivity. This may encourage the individual development of pharmacists working in these pharmacies.

Lastly, the implementation of these competency frameworks worldwide has raised concerns about their inappropriate use; often, they have been used as ticking boxes, and more time has been spent on bureaucratic requirements than on individual development [19]. Subsequently, competency frameworks have not been utilised as expected, and pharmacists are not always motivated to assess their competencies. Given the high response rate in this study (85.8%), the authors believe that the community pharmacist-preceptors included in this analysis were highly motivated because they perceived the multi-purpose use of CCF; it not only enabled them to identify their learning needs and supported their individual development, but assisted in getting better acquainted with the competency-based training programme. As a result, they were able to get a better understanding of their mentor roles. Moreover, pharmacist-preceptors were required to assess students’ competencies at the end of the 6-month pre-registration training, and, with CCF being a new concept, they were aware that initial self-assessment would be important if they were to assess students. Nash et al. examined professional Competency Standards in Australia with regard to extent of use and perceived relevance. Their findings imply that practicing pharmacists did not appear to be utilising the National Competency Standards although these were mandatory. In contrast, pharmacists in preceptor roles stated that their practical and repeated application of the National Competency Standards with their interns increased their own use of competency standards in CPD [20]. Our findings confirmed that application of the competency framework was important for pharmacist-preceptors and their role in student training.

There were several limitations of this study. Firstly, the participants of the study were those who volunteered to act as mentors, therefore we could assume that they are not representative of general pharmacy population and the results could not be transferable to the pharmacist population in Croatia, as a whole. Despite this, based on demographic characteristics of the sample (ie. gender distribution, years of experience, type of pharmacy etc.) our sample compares to the general community pharmacists population. Second, this study used self-assessment of performance as an outcome measure and it is well know that there are many influences on self-assessment that can lead to unreliable ratings [2, 4].

Mindful of the above mentioned limitations, this study does help to define areas where additional professional education may be required. Based on these results, we suggest that continuing education providers in Croatia should focus on areas where community pharmacists are deficient, ie. public health and assurance of safe medicines use in particular. Moreover, educators should put an effort in rethinking the instructional design which will enable the development of competences as opposed to providing only knowledge-based courses which are offered at the moment. The findings of this study may serve as a guide for creating more appropriate mentor training programmes, which will emphasise the development of competencies necessary for providing higher quality pharmacy services.

## Conclusion

This research represents the first analysis of the CCF in practice and has identified that community pharmacist-preceptors perceived themselves to be most competent in the organization and management competencies. However, while they expressed a high degree of competence in tasks related to medicine dispensing, they considered themselves lacking in some competencies in the “Pharmaceutical Care” and “Pharmaceutical Public Health” clusters. Moreover, younger pharmacists with a postgraduate qualification who worked for large pharmacy chains in the capital area and who had been in their current posts for a shorter period of time perceived themselves to be more competent. This study’s findings could help to define areas in which community pharmacist-preceptors may need additional professional education. Consequently, could help to develop action planning for improvements in continuing education as well as developing better pharmacy student training programme.

## Abbreviations

ADR(s): adverse drug reaction(s)
CCF: Croatian Competency Framework
CE: continuing education
CPD: continuing professional development
FIPEd: International Pharmaceutical Federation Education
GbCF: Global Competency Framework
GLF: General Level Framework; UoZ University of Zagreb
